# Large-Scale Contractility Measurements Reveal Large Atrioventricular and Subtle Interventricular Differences in Cultured Unloaded Rat Cardiomyocytes

**DOI:** 10.3389/fphys.2020.00815

**Published:** 2020-07-21

**Authors:** Edgar E. Nollet, Emmy M. Manders, Max Goebel, Valentijn Jansen, Cord Brockmann, Jorrit Osinga, Jolanda van der Velden, Michiel Helmes, Diederik W. D. Kuster

**Affiliations:** ^1^Department of Physiology, Amsterdam UMC, Vrije Universiteit Amsterdam, Amsterdam Cardiovascular Sciences, Amsterdam, Netherlands; ^2^CytoCypher BV, Wageningen, Netherlands

**Keywords:** cardiomyocyte, contractility, large-scale, atria, ventricles, regional differences

## Abstract

The chambers of the heart fulfill different hemodynamic functions, which are reflected in their structural and contractile properties. While the atria are highly elastic to allow filling from the venous system, the ventricles need to be able to produce sufficiently high pressures to eject blood into the circulation. The right ventricle (RV) pumps into the low pressure pulmonary circulation, while the left ventricle (LV) needs to overcome the high pressure of the systemic circulation. It is incompletely understood whether these differences can be explained by the contractile differences at the level of the individual cardiomyocytes of the chambers. We addressed this by isolating cardiomyocytes from atria, RV, LV, and interventricular septum (IVS) of five healthy wild-type rats. Using a high-throughput contractility set-up, we measured contractile function of 2,043 cells after overnight culture. Compared to ventricular cardiomyocytes, atrial cells showed a twofold lower contraction amplitude and 1.4- to 1.7-fold slower kinetics of contraction and relaxation. The interventricular differences in contractile function were much smaller; RV cells displayed 12–13% less fractional shortening and 5–9% slower contraction and 3–15% slower relaxation kinetics relative to their LV and IVS counterparts. Aided by a large dataset, we established relationships between contractile parameters and found contraction velocity, fractional shortening and relaxation velocity to be highly correlated. In conclusion, our findings are in line with contractile differences observed at the atrioventricular level, but can only partly explain the interventricular differences that exist at the organ level.

## Introduction

The heart consists of atria and ventricles which at a cellular level are activated through excitation–contraction coupling in a highly coordinated fashion to provide sufficient pressure to maintain perfusion of the body. The structural and contractile properties of each compartment are linked to the hemodynamic function it fulfills. The right ventricle (RV) ejects blood into the low pressure pulmonary vasculature, whereas the left ventricle (LV) pumps blood into the high pressure systemic circulation. As a result, the wall of the RV is considerably thinner and more compliant than the LV wall (Voelkel et al., 2006). The atria function predominantly as a reservoir for venous return and under normal circumstances does not have to overcome high pressures during contraction, which is reflected by highly elastic properties of the atrial myocardium (Blume et al., 2011). Functional differences between the chambers of the heart are also apparent in the differential response to changes in hemodynamics, such as chronic pressure overload (Belin et al., 2011; Rain et al., 2014).

The structural and functional differences between the chambers of the heart may be explained by distinct functional properties of individual cardiomyocytes from each region. Up to date, the contractile function of cardiomyocytes from different regions has not been subject of intensive study. The limited number of studies covering this topic reports inconclusive findings (McMahon et al., 1996; Tanaami et al., 2005; Kondo et al., 2006; Sathish et al., 2006; Belin et al., 2011; Chu et al., 2013), which may be attributed to species differences, methodological differences, selection bias, and small sample sizes (Chung and Campbell, 2013; Sikkel et al., 2017; Clark and Campbell, 2019; Kuster, 2019). Furthermore, regional differences may be small and can therefore only be studied through unbiased, extensive sampling.

We performed large-scale contractility measurements on intact unloaded rat cardiomyocytes isolated from atrial (AT), LV, RV, and interventricular septal (IVS) tissue to assess whether differences in contractile properties exist at the level of single cardiomyocytes.

## Materials and Methods

### Ethical Approval

The animal experiments were performed in accordance with the guidelines from Directive 2010/63/EU of the European Parliament on the protection of animals used for scientific purposes and were approved by the ethics committees of VU University Medical Center, Amsterdam, Netherlands.

### Adult Rat Cardiomyocyte Isolation and Culturing

Cardiomyocytes were isolated from adult Wistar rats (*n* = 5) weighing 200–250 g. Intact AT (left and right atrium were combined), LV, RV, and IVS rat cardiomyocytes were isolated through collagenase digestion of hearts essentially as described previously (Sequeira et al., 2015; van Deel et al., 2017). In brief, rats were euthanized via isoflurane inhalation, after which hearts were quickly harvested and washed in cold isolation Tyrode solution (130 mM NaCl, 5.4 mM KCl, 3 mM sodium pyruvate, 25 mM HEPES, 0.5 mM MgCl_2_, 0.33 mM NaH_2_PO_4_, 22 mM glucose, pH 7.4) containing 0.2 mM EGTA (Tyrode–EGTA). Hearts were subsequently cannulated to a Langendorff set-up via the aorta and perfused with Tyrode-EGTA for 2 min at 37°C. Next, hearts were perfused for 25–35 min (depending on the integrity of the tissue) with enzyme Tyrode solution composed of Tyrode solution, 50 μM CaCl_2_, and 1.2 mg/mL collagenase (Type II, 265 U/mg; Worthington Biochemical, NJ, United States). The heart was removed from the cannula and separated into AT, LV, RV, and IVS. Each part was cut into fine pieces, followed by trituration for 3 min with a plastic Pasteur pipette in stopping buffer solution 1 [SB-1; Tyrode solution, 0.6 mg/mL collagenase, 100 μM CaCl_2_, and 10 mg/mL bovine serum albumin (BSA)]. Cell suspensions were filtered through a 300 μm nylon mesh filter and collected in 50 mL Falcon tubes, followed by centrifugation for 1 min at 27 × *g* at room temperature. Pellets containing cardiomyocytes were resuspended in SB solution 2 (SB-2; Tyrode solution, 250 μM CaCl_2_ and 10 mg/mL BSA) and incubated for 10 min at 37°C in order for the cells to settle. After the removal of supernatants, cardiomyocytes were resuspended in SB solution 3 (SB-3; Tyrode solution, 500 μM CaCl_2_ and 10 mg/mL BSA) and incubated for 10 min at 37°C. Hereafter, cardiomyocytes were suspended in plating medium [Medium 199 (Lonza, Basel, Switzerland), 1% penicillin/streptomycin and 5% fetal bovine serum (FBS)] and transferred to laminin-coated 35 mm^2^ glass-bottomed dishes (MatTek, Ashland, MA, United States). Following 1 h of incubation at 37°C in humidified air with 5% CO_2_, unattached cells were washed away by replacing plating medium with culture medium [Medium 199, 1% penicillin/streptomycin, insulin transferrin selenium (ITS; Sigma-Aldrich, composition: insulin, 10 mg/L; transferrin, 5.5 mg/L; selenium 5 μg/L) and 0.5 μM cytochalasin D (Life Technologies)]. The latter two compounds were added in order to prevent dedifferentiation and optimize cell viability (Viero et al., 2008; Tian et al., 2012). Cardiomyocytes were cultured overnight in an incubator at 37°C in humidified air with 5% CO_2_.

### Cardiomyocyte Contractility

**FIGURE 1:**
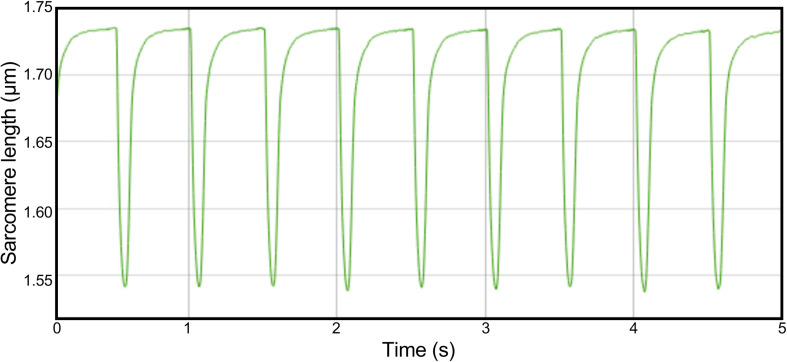
Example trace of a full-length recording in a single cell. Cells were paced with a frequency of 2 Hz. Sarcomere length was measured for 5 s, in which 8 or 9 contractions could be recorded.

Cardiomyocyte contractility measurements were performed using the CytoCypher Multicell High Throughput System (CytoCypher BV, Wageningen, Netherlands). To measure contractility, culture medium was replaced by experimental Tyrode solution (137 mM NaCl, 5.4 mM KCl, 3 mM sodium pyruvate, 5 mM HEPES, 0.57 mM MgCl_2_, 0.33 mM NaH_2_PO_4_, 1.8 mM CaCl_2_ and 5.6 mM glucose, pH 7.4). Dishes were placed on a fast *x*–*y*–*z* position programmable scanning microscope, allowing rapid identification and measurements of contracting cardiomyocytes. Cell shortening experiments were performed as described previously (Juni et al., 2019). Contraction was evoked via electrical field stimulation (2 Hz, 4 ms pulse duration, 25 V) at 37°C. Sarcomere shortening and relaxation kinetics were quantified via a video-based sarcomere length (SL) detection system (IonOptix corporation, Milton, MA, United States) at 250 Hz sampling frequency. Each cell was measured for 5 s, in which 8–9 contraction traces could be recorded (see Figure 1 for an example of a full-length trace of a single cell). All contracting cardiomyocytes that could be measured within a 20 min time frame per dish were measured. We observed no significant rundown in contractility during this period. In a subset of cells, cardiomyocyte width and length were assessed via edge detection. Width was measured at three locations along the length of the cell (at 25, 50, and 75%). Contractility assays were performed within 18–24 h after isolation. During this period, no decline in contractility was observed.

### Data Analysis

**FIGURE 2:**
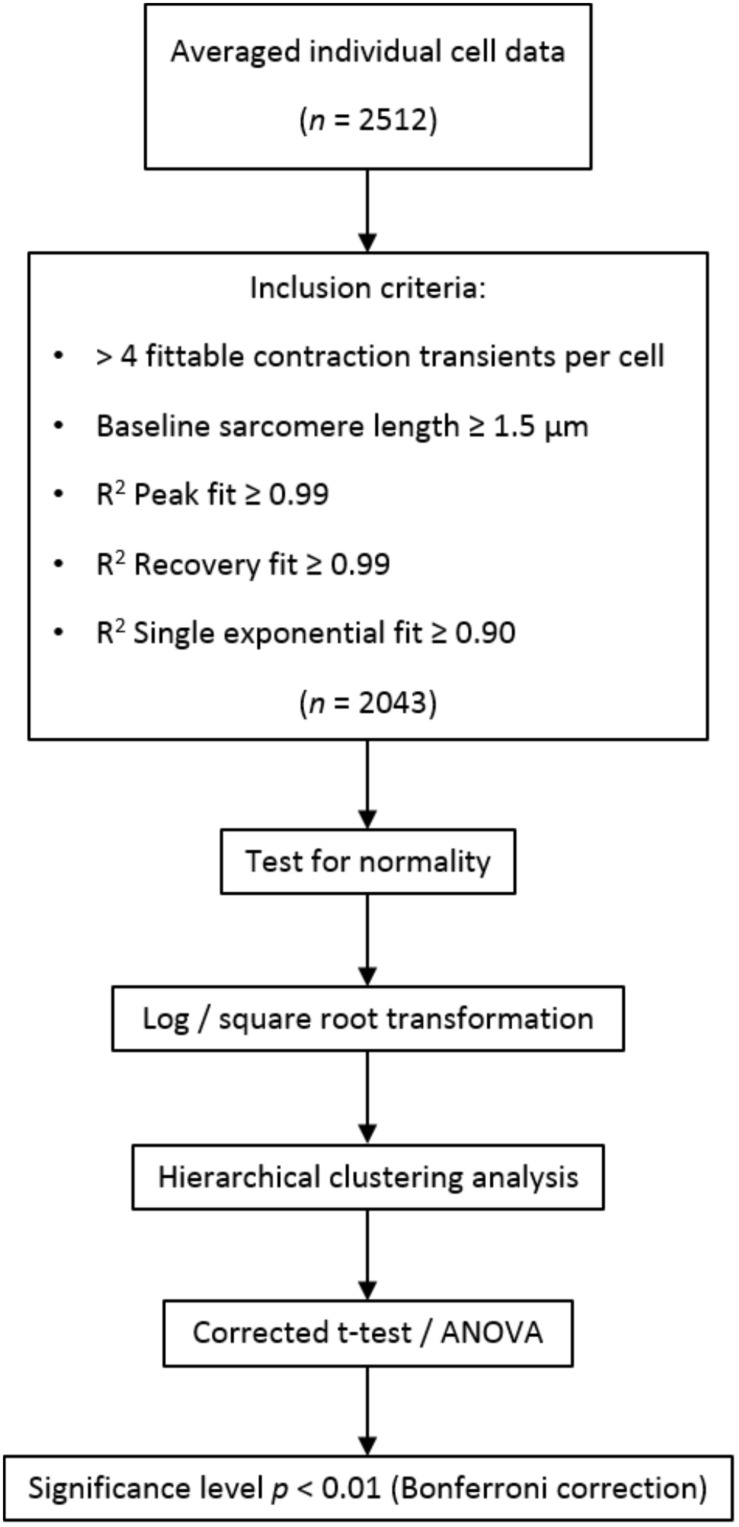
Schematic overview of data processing and analysis. In total, 2,512 cells were measured, of which 2,043 were included in analysis. If no more than four contraction transients could be fitted by the transient analysis software (i.e., due to disturbances in the sarcomere length signal), cells were excluded from the analysis. Short resting sarcomere length and inaccurate fitting of the contraction transients were additional exclusion criteria.

A schematic overview of data analysis is depicted in Figure 2. The batch of raw contractility data was analyzed using the CytoSolver Transient Analysis Tools package from CytoCypher BV (Wageningen, Netherlands) to yield averaged contractile and kinetic parameters from each cell. With this software, the relaxation phase is fitted with a bi-exponential fit starting at 10% return time. Individual cells were excluded from further analysis if they did not meet all inclusion criteria (Figure 2). Data were subsequently tested for normality and transformed via log or square root transformation when appropriate. Hierarchical clustering analysis (Sikkel et al., 2017) was performed to quantify the amount of clustering for each rat and dish. If the data were tightly grouped, appropriate corrections to the statistical significance test were applied. A student’s *t*-test or two-way analysis of variance (ANOVA) where appropriate was performed to test for differences between regions, followed by Bonferroni *post hoc* testing. The significance level was set at *p* < 0.01.

## Results

### Contractility and Cell Dimension Differences Between Heart Regions

**FIGURE 3:**
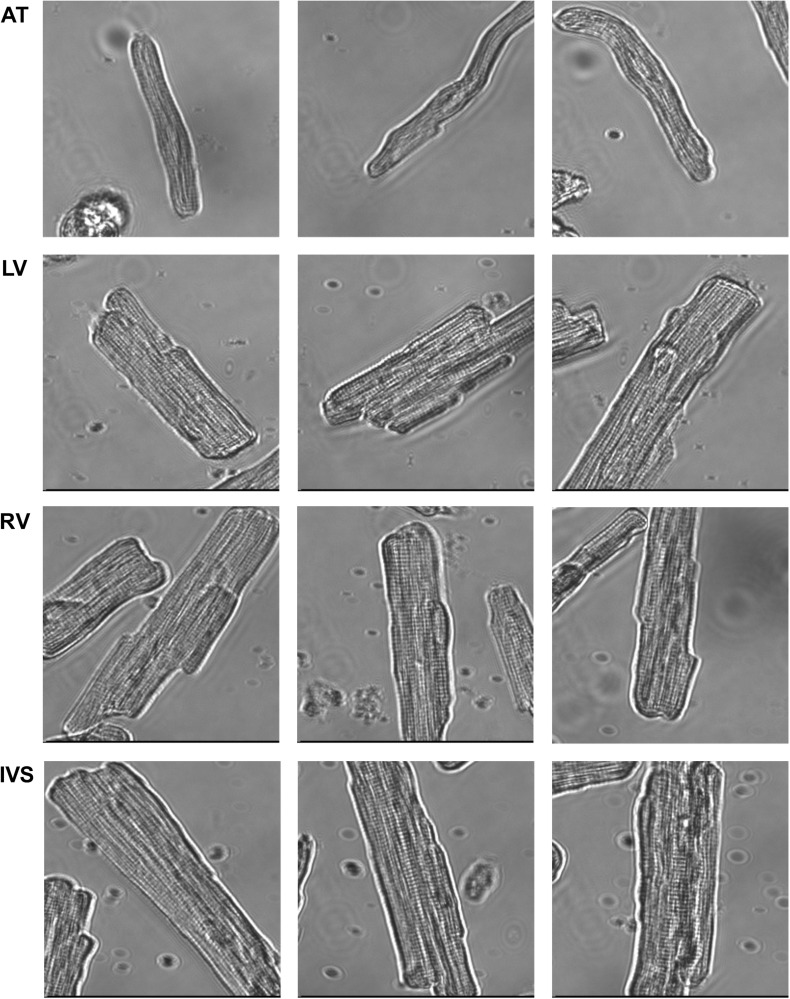
Representative cells from each cardiac region. Cells from atrium (AT) were markedly thinner and shorter than cells from left ventricle (LV) and right ventricle (RV) and interventricular septum (IVS).

**FIGURE 4:**
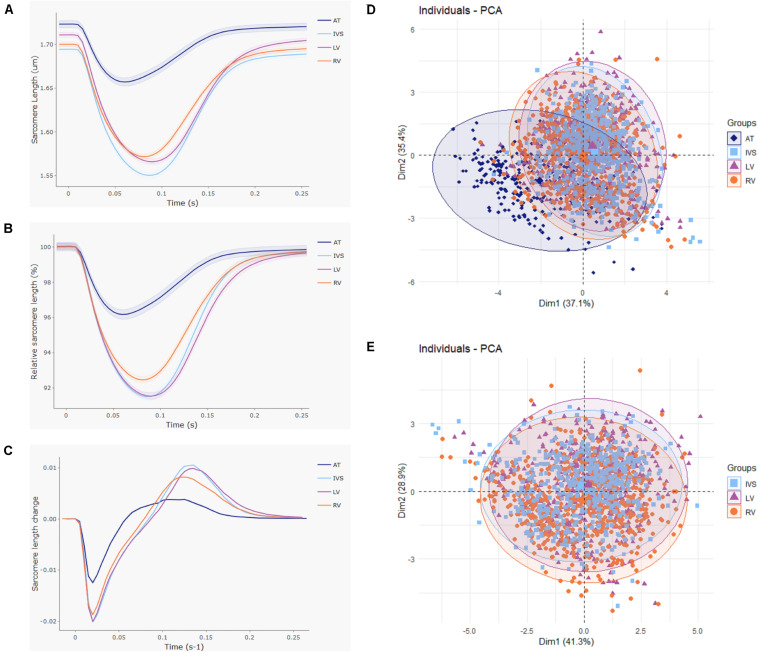
Large atrioventricular and small interventricular differences in contractile function. Contractile function was compared between cardiomyocytes isolated from atria (AT; *n* = 272), left (LV; *n* = 553), and right ventricle (RV; *n* = 711) and interventricular septum (IVS; *n* = 507) from five rats. **(A)** Averaged and **(B)** normalized contraction traces and **(C)** averaged velocity traces for each cardiac region. Shading indicates SEM. **(D)** Principal component analysis displays clear clustering of AT cells. **(E)** Principal component analysis with AT cells omitted shows no separate clusters at the ventricular level.

In total, 2,512 cardiomyocytes from five healthy rats were measured, of which 2,043 met the inclusion criteria for further analysis (Figure 2). Representative cells from each region are shown in Figure 3. Averaged contraction and velocity traces of each region are depicted in Figures 4A–C. These traces indicate markedly different contraction characteristics in AT cells compared to cells isolated from ventricular regions. A principal component analysis (PCA) was carried out to assess if cardiomyocytes from AT, LV, RV, and IVS display clustering based on their respective origin (Figure 4D). Hereto, redundant variables (e.g., “Time to Peak 10%,” “Time to Peak 20%”) were omitted to avoid overrepresentation of similar variables in the dimension axes. This confirmed that only cells from AT were clearly distinct from cells from the ventricular regions. To elucidate, the main purpose of a PCA is to reduce the number of variables in a dataset, especially when many variables are correlated with each other, while retaining the variation and differences in the dataset (Jolliffe and Cadima, 2016). Hence, the variables are “combined” mathematically into several dimensions that indicate the percentage of differences retained. In our case, Dim 1 represents 37% of the difference in the dataset, while Dim 2 represents 35%. In Supplementary Figure S1, the length of the arrow indicates how strongly the parameter affects the dimension. Thus, fractional shortening, relaxation velocity, contraction velocity, and time to peak all contribute substantially to the difference between AT and ventricular cells. Excluding the AT cells from the analysis showed that ventricular cells do not display clustering based on regional origin (Figure 4E).

To further address how contractility of AT cells differ from ventricular cells, data from LV, RV, and IVS cells were pooled (labeled “VT”) and tested for significance against AT cells after correcting for normality and variation arising from rat and dish differences (Table 1). Cells from AT tissue were found to differ strongly from VT cells for every parameter tested except 80% relaxation time and relaxation coefficient tau. Most notably, fractional shortening in AT cells was twofold lower compared to VT cells. Velocities of contraction and relaxation were substantially lower in AT cells compared to values observed in VT cardiomyocytes. Despite lower contraction velocity, AT cells reached peak earlier than VT cells. Similarly, partial relaxation is achieved earlier in AT cells than VT cells, in spite of lower maximal relaxation velocity in AT cells. In terms of cell dimensions, AT cells were found to be both shorter and smaller compared to VT cells, indicating that, as anticipated, atrial cardiomyocytes are both structurally and functionally distinct from ventricular cardiomyocytes.

**TABLE 1:** Contractile and cell dimension differences between AT and ventricular (VT) cells.

**Contractility parameters**	**AT**	**VT**	
	***n* = 272**	***n* = 1771**	
Baseline SL (μm)	1.720.07	1.700.07	*
Peak height (μm)	0.070.05	0.140.05	**
Fractional shortening (%)	4.33.0	8.42.6	**
Time to peak (ms)	55.017.8	84.120.1	**
Time to peak 80 (ms)	31.28.7	44.29.8	**
Time to peak 50 (ms)	20.75.2	26.86.2	**
Time to peak 20 (ms)	13.13.2	15.83.9	**
Contraction velocity (μm/s)	−3.42.6	−4.62.0	**
80% Relaxation time (ms)	61.031.6	63.318.9	
50% Relaxation time (ms)	34.014.5	43.613.8	**
20% Relaxation time (ms)	18.88.6	28.410.3	**
Relaxation velocity (μm/s)	2.11.8	3.51.7	**
Tau (ms^–1^)	37.130.8	36.223.8	

**Cell dimensions**	**AT**	**VT**	
	**Width: *n* = 106**	**Width: *n* = 982**	
	**Length: *n* = 63**	**Length: *n* = 633**	

Cell width (μm)	22.77.9	32.27.0	**
Cell length (μm)	80.826.1	98.917.7	**

Data are illustrated as mean ± SD. Significant differences are indicated by **p < 0.001 or *p < 0.01 versus AT.

To define if differences between RV and LV structure and function arise from inherent changes in the cardiomyocytes that make up the ventricles, we compared dimensions and contractile properties of RV, LV, and IVS cardiomyocytes (Table 2). RV cells were found to have a lower amplitude of contraction than LV and IVS when expressed both in absolute terms (peak height) as well as relative terms (fractional shortening). With respect to contraction kinetics, RV cardiomyocytes showed a slightly lower velocity of shortening compared to LV and IVS. Relaxation velocity in IVS cells was higher compared to RV and LV cells. Compared to LV, a trend toward lower relaxation velocity was observed in RV (*p* = 0.02). RV cells moreover reached peak earlier than LV and IVS cells. Lastly, with respect to cell dimensions, LV cardiomyocytes were larger both in terms of width and length compared to RV. The analysis on regional differences in fractional shortening and contraction and relaxation velocities were performed on cardiomyocytes from five animals taken together, but can also be observed when looking at individual animals (Supplementary Figure S2).

**TABLE 2:** Interventricular contractile and cell dimension differences.

**Contractility parameters**	**LV**			**RV**		**IVS**
	***n* = 553**			***n* = 711**		***n* = 507**
Baseline SL (μm)	1.710.07			1.700.07		1.700.08
Peak height (μm)	0.150.04	**		0.130.05	††	0.150.04
Fractional shortening (%)	8.72.6	**		7.82.7	††	8.82.5
Time to peak (ms)	88.420.5	*		80.020.0	††	85.218.9
Time to peak 80 (ms)	45.610.0	*		42.59.5	††	45.19.6
Time to peak 50 (ms)	27.46.3	*	#	25.75.8	††	27.66.4
Time to peak 20 (ms)	16.14.0		#	15.03.5	††	16.64.2
Contraction velocity (μm/s)	−4.61.9	*		−4.42.1	†	−4.82.0
80% Relaxation time (ms)	67.518.9		#	62.720.3		59.516.0
50% Relaxation time (ms)	46.713.9			42.814.5		41.312.2
20% Relaxation time (ms)	30.510.3			27.710.6		27.19.4
Relaxation velocity (μm/s)	3.41.6		#	3.31.7	††	3.81.7
Tau (ms^–1^)	38.225.2			34.921.4		35.925.2

**Cell dimensions**	**LV**			**RV**		**IVS**
	**Width: *n* = 337**			**Width: *n* = 379**		**Width: *n* = 266**
	**Length: *n* = 210**			**Length: *n* = 259**		**Length: *n* = 164**

Cell width (μm)	33.57.4	**		31.16.6		32.36.9
Cell length (μm)	102.019.0	*		97.718.0		97.014.8

Data are illustrated as mean ± SD. Significant differences are indicated by *p < 0.01 or **p < 0.001 for LV versus RV; ^#^*p* < 0.01 or ^##^*p* < 0.001 for LV versus IVS; ^†^*p* < 0.01 or ^††^*p* < 0.001 for RV versus IVS.

### Correlation Between Contractility Parameters

**FIGURE 5:**
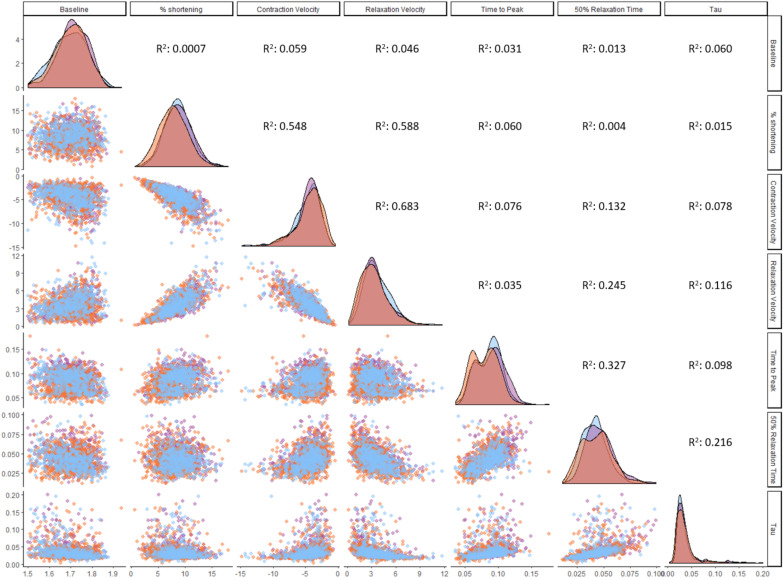
Correlations between contractility parameters. Correlation analysis was performed on ventricular cardiomyocytes (*n* = 1,771). Distribution plots and individual cardiomyocytes are colored based on their origin: RV (orange), LV (purple), and IVS (blue). All correlations with an *R*^2^ larger than 0.01 are statistically significant at the level of *p* < 0.001.

The size of our dataset allowed us to establish correlations between contractility parameters (Figure 5). Contraction velocity strongly correlates with relaxation velocity (*R*^2^ = 0.68). Fractional shortening displays robust correlation with both contraction velocity (*R*^2^ = 0.55) and relaxation velocity (*R*^2^ = 0.59), but correlates poorly with time to peak (*R*^2^ = 0.06). No regional differences were observed in correlations between these parameters when RV, LV, and IVS cells were analyzed separately (data not shown). Fractional shortening shows no correlation with relaxation time (50% relaxation time; *R*^2^ = 0.004), and relaxation coefficient (tau; *R*^2^ = 0.06). These findings imply that contraction velocity is the most important determinant of the degree of contraction and the velocity of subsequent relaxation.

### Relation Between Effect Size and Sample Size

**FIGURE 6:**
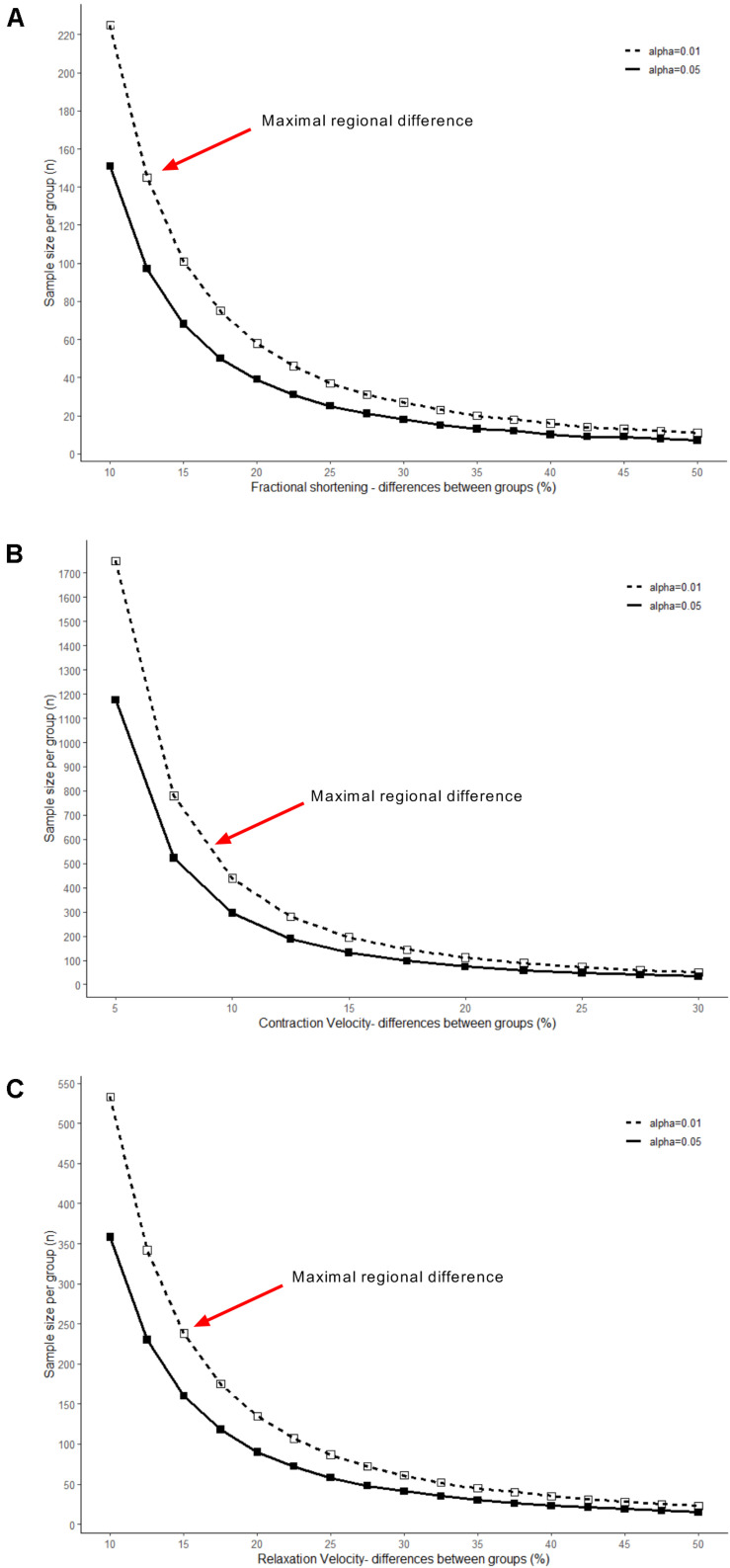
Relationships between effect and sample size. Sample sizes needed to detect a range of effect sizes were calculated and plotted, using the variability of ventricular cells in: **(A)** fractional shortening, **(B)** contraction velocity, and **(C)** relaxation velocity. Solid line shows effect size at significance level of *p* < 0.05, dashed line of *p* < 0.01. Red arrows indicate sample size required for uncovering effect sizes at the magnitude of the regional differences identified in this study.

The large-scale dataset obtained in this study provides an accurate estimation of the variation that is present among ventricular cardiomyocytes. Therefore, the standard deviations of the parameters reported in this study (Tables 1, 2) can be used to calculate the sample size that is needed to uncover an anticipated effect size. Calculating the sample size for a range of effect sizes generates an exponential function, which is exemplified in Figure 6 for fractional shortening, contraction velocity, and relaxation velocity. When subtle differences are subject of study, such as the regional differences reported here, a large sample size is required, in particular when kinetics of contraction are addressed. To elucidate, fractional shortening in RV cells was 7.8%, and in IVS cells, it was 8.8%. Hence, this constitutes an effect size of 13% and requires a sample size of 140 per group in order to be detected with statistical significance at the level of *p* < 0.01. In contrast, β-adrenergic receptor stimulation with isoproterenol typically induces effect sizes of more than 50% (Harding et al., 1988; Najafi et al., 2016) and hence can be studied using relatively small sample sizes (*n* = 10–20). This may serve as guidance to future studies investigating contractility differences between (experimental) conditions.

## Discussion

In the present study, we deployed a high-throughput system to measure contractile properties and cell dimensions in AT, LV, RV, and IVS cardiomyocytes isolated from healthy rats. We observed major differences between AT and ventricular cells. Moreover, we report relatively small, but significant contractile and dimensional differences between cardiomyocytes from ventricular regions, most notably lower contraction amplitude and kinetics in RV cardiomyocytes compared with LV and IVS. Differences between atrial and ventricular cardiomyocytes are in keeping with the respective functions of atria and ventricles. The much smaller differences between RV, IVS, and LV cardiomyocytes can only partly explain the differences between RV and LV that exist at the organ level. Our large dataset enabled us to detect small differences despite the remarkable heterogeneity we demonstrate to be present among cells. We could also correlate contractile parameters, revealing a strong correlation between contraction velocity, fractional shortening, and relaxation velocity. Taken together, our study highlights the importance of extensive, unbiased sampling when performing studies on cardiomyocyte contractility.

### Contractile Differences Between AT and Ventricular Cardiomyocytes

To the best of our knowledge, unloaded shortening in AT cardiomyocytes relative to ventricular cells has not been comprehensively assessed. A study by Tanaami et al. (2005) reports no contractile differences between rat AT and VT cardiomyocytes in terms of fractional shortening. However, this was based on experiments in a small number of cardiomyocytes (*n* = 12–30) in which a large standard error was observed. Under loaded conditions, Luss et al. (1999) demonstrated AT cells to reach peak force development in a shorter time period than LV cells, which is in good agreement with our observation that AT cells achieve peak shortening earlier than ventricular cells (Table 1). This might in part be explained by higher expression of the fast α-isoform of cardiac myosin heavy chain (MHC) and faster cross-bridge kinetics in atrial relative to ventricular tissue (Reiser and Kline, 1998; Andruchov et al., 2006), although in the present study contraction velocity was lower in atrial than in ventricular cardiomyocytes. Relative to ventricular cells, AT cells display shorter action potentials, more heterogeneous intracellular Ca^2+^ transient propagation, and higher sarcoplasmic reticulum (SR) Ca^2+^-ATPase (SERCA2) levels (Minajeva et al., 1997; Luss et al., 1999; Walden et al., 2009; Greiser et al., 2014); hence, Ca^2+^-release from the SR and subsequent cross-bridge activation may be less uniformly orchestrated, which combined with faster Ca^2+^ re-uptake may give rise to short-lived, relatively slow contractions (Brandenburg et al., 2016). This would explain the low amplitude of fractional shortening and slow kinetics of contraction observed in our study. Faster Ca^2+^ re-uptake may also in part explain why in our study AT cells required less time to re-lengthen during the early phase of relaxation, although lower relaxation velocity indicates overall slower relengthening than ventricular cells.

### Contractile Differences Between Ventricular Regions

Our findings predominantly suggest that RV cardiomyocytes show reduced contraction amplitude and kinetics of contraction and relaxation compared to LV and IVS. Hence, under unloaded conditions cardiomyocytes display similarity to RV and LV contraction *in vivo*, i.e., less shortening and slower relaxation as assessed by tricuspid and mitral valve inflow in RV versus LV (Zoghbi et al., 1990; Pela et al., 2004; Hudsmith et al., 2005). Comparing shortening velocity under unloaded conditions to tissue contraction velocity is complicated due to differences in fiber orientation, which is more longitudinal in the RV and more circular in the LV (Naito et al., 1995). Accordingly, in the RV, longitudinal shortening is the main contributor to contraction, as opposed to circumferential shortening in the LV.

Our general finding of lower contractility in the RV versus LV is consistent with other studies reporting interventricular differences in unloaded shortening in rodent cardiomyocytes (Kondo et al., 2006; Sathish et al., 2006; Chu et al., 2013). However, the percent difference in shortening between LV and RV cells in these studies is considerably larger (ranging from 25 to 80%) than the difference we report here (12%). A similar trend is seen for kinetics of contraction, where we measured a 5% difference in contraction velocity between RV and LV, compared to 44% in the study by Kondo et al. (2006). Importantly, we measured shortening at a tightly controlled physiological temperature (37°C), whereas comparable experiments were carried out at less optimally controlled room temperature (19–22°C) (Kondo et al., 2006; Sathish et al., 2006; Chu et al., 2013). Cross-bridge cycling and concomitant cardiomyocyte shortening are highly temperature sensitive (de Tombe and Stienen, 2007; Chung and Campbell, 2013); thus, any differences that may exist between RV and LV cells could be magnified when measuring at non-physiological temperatures. Also, we detected no LV versus RV difference in relaxation as defined by the time constant tau, dissimilarly to Chu et al. (2013) who report a twofold higher value in RV versus LV. This discrepancy might be explained by different experimental temperatures, as this heavily influences cardiomyocyte relaxation (Chung and Campbell, 2013). A study by McMahon et al. (1996) addressing unloaded shortening in large numbers of porcine cardiomyocytes reported higher contractility in RV compared to LV cells, which is in contrast to findings in rodents. As these data were gathered under experimental conditions identical to our study, their findings likely reflect a species difference.

The exact mechanisms underlying the interventricular differences reported here remain largely enigmatic. The RV has a different embryological origin than the LV (Cai et al., 2003); hence, contractile differences may simply be a reflection thereof. Additionally, the LV and RV may adapt to loading conditions not only structurally via cardiomyocyte count but also at the level of individual cells. The latter is also reflected in the cell dimension difference we observed, with LV cells being on average larger than RV cells. Findings from proteomic studies indicate LV versus RV differences in protein expression levels of sarcomere proteins such as myosin light chain 2, α-MHC and cardiac myosin binding protein C, and tropomyosin- and titin isoforms, i.e., more stiff titin in the LV (Comunian et al., 2011; Cadete et al., 2012; Peng et al., 2013). These differences may partly contribute to the contraction properties of LV and RV myofilaments described by Belin et al. (2011), in addition to the differences in myofilament phosphorylation they observed. Titin isoform composition could play a role in the kinetics and extent of contraction, as we recently showed (Najafi et al., 2019). Furthermore, LV versus RV differences have been found in terms of ion channel make-up and regulation (Gaborit et al., 2007; Molina et al., 2016; Zaitsev et al., 2019). As the IVS separates the LV and RV and is formed from the cell populations of both LV and RV embryological origin in rodents (Franco et al., 2006), we expected IVS cells to have contractile parameters intermediate between RV and LV. However, there were very few significant differences between LV and IVS, but almost all measured parameters were different between IVS and RV. This indicates that with respect to contraction/relaxation, IVS and LV are more similar to each other than the RV. Taken together, LV and RV are distinct from one another in multiple cellular aspects, which may provide a substrate for contractile differences and represents an interesting avenue for future research.

### Intraregional Variability

We observed remarkable intraregional variation in cardiomyocyte contractility, which may stem from several factors. For example, Clark and Campbell (2019) recently demonstrated that variation in unloaded rat cardiomyocytes decreases if cells are sampled from a smaller area, implying that local stretch and strain and paracrine signaling impact on cellular contractility (Kuster, 2019). Heterogeneity may also arise from differences between transmural regions. Transmural differences have been described for numerous properties, such as length-dependent activation, action potential waveforms, calcium homeostasis, mitochondrial respiration, ion channel make-up, and myofilament protein phosphorylation (Gaborit et al., 2007; Cazorla and Lacampagne, 2011; Gregorich et al., 2015; Kindo et al., 2016), which may all influence contractility. Experiments in a limited number of unloaded cardiomyocytes from subepicardial, midmyocardial and subendocardial regions suggest the existence of subtle contractile differences (Chung and Campbell, 2013), which would be an interesting subject for more extensive study.

### Correlations Between Contractile Parameters

The large scale of our dataset enabled us to look for correlations between different parameters. We found a strong correlation between contraction velocity, fractional shortening, and relaxation velocity. The correlation between relaxation velocity and amplitude of shortening was previously described to occur in canine (Tsutsui et al., 1993) and porcine cardiomyocytes (McMahon et al., 1996). Also in a recent paper by Clark and Campbell (2019), a correlation was observed between cell shortening and time to 50% relaxation in rat cardiomyocytes. Cells that showed a greater amplitude of cell shortening had a shorter relaxation time. In the current study, the velocity of relaxation correlated much better to shortening than time to 50% relaxation. This might be expected as relaxation time is more dependent on the amplitude of contraction than velocity. The observation that there is a robust correlation between contraction and relaxation velocity was shown in an elegant paper by Janssen (2010). Here he compiled data from isometric contractions of mouse trabeculae that his group had gathered over the years and showed that under a wide variety of conditions, contraction and relaxation velocities correlated very strongly. The relationship between kinetics of contraction and relaxation seemed to be a property of the myofilaments, as perturbations to calcium transients did not influence the correlation (Janssen, 2010). The exact mechanism that determines this relationship is not well understood, but could involve cardiac myosin binding protein C (Janssen, 2010), which is an important mediator of cross-bridge cycling (Previs et al., 2014; Sequeira et al., 2014).

### Limitations

We acknowledge several methodological limitations in this study. We combined cardiomyocytes from the left and right atrium in order to obtain a sufficient number of atrial cells, thus obscuring contractile differences that may exist between cardiomyocytes from these two regions. Furthermore, measurements were performed after overnight culture, allowing cells to recover from stress and damage induced during isolation (Mitcheson et al., 1998) and enabling us to perform all individual animal measurements on a single day. However, during this culturing period, regional contractile differences may become less pronounced compared to that directly after isolation.

## Conclusion

Here, we report large atrioventricular contractile differences that are in line with differences at the organ level. The interventricular differences we found are more subtle and reflect only in part the differences observed *in vivo*. Through extensive sampling we furthermore identified strong correlations between contraction velocity, fractional shortening and relaxation velocity. Lastly, our large-scale dataset demonstrates notable intraregional heterogeneity, providing valuable methodological insights for future cardiomyocyte shortening studies.

## Data Availability Statement

All datasets generated for this study are available upon request.

## Ethics Statement

The animal study was reviewed and approved by the ethics committees of the VU University Medical Center, Amsterdam, Netherlands.

## Author Contributions

EN, EM, MH, and DK designed the study, wrote the manuscript, and performed the experiments and data analysis. MG, VJ, CB, and JO helped performing the experiments and edited the manuscript. JV helped drafting and revising the manuscript. All authors contributed to the article and approved the submitted version.

## Conflict of Interest

EM is an employee of CytoCypher BV. MH is CEO of CytoCypher BV. The remaining authors declare that the research was conducted in the absence of any commercial or financial relationships that could be construed as a potential conflict of interest.
