# Supplementary figures and images for: Large-Scale Contractility Measurements Reveal Large Atrioventricular and Subtle Interventricular Differences in Cultured Unloaded Rat Cardiomyocytes
Source: Front Physiol. 2020 Jul 21;11:815. doi: 10.3389/fphys.2020.00815 (PMC7396550; doi:10.3389/fphys.2020.00815)

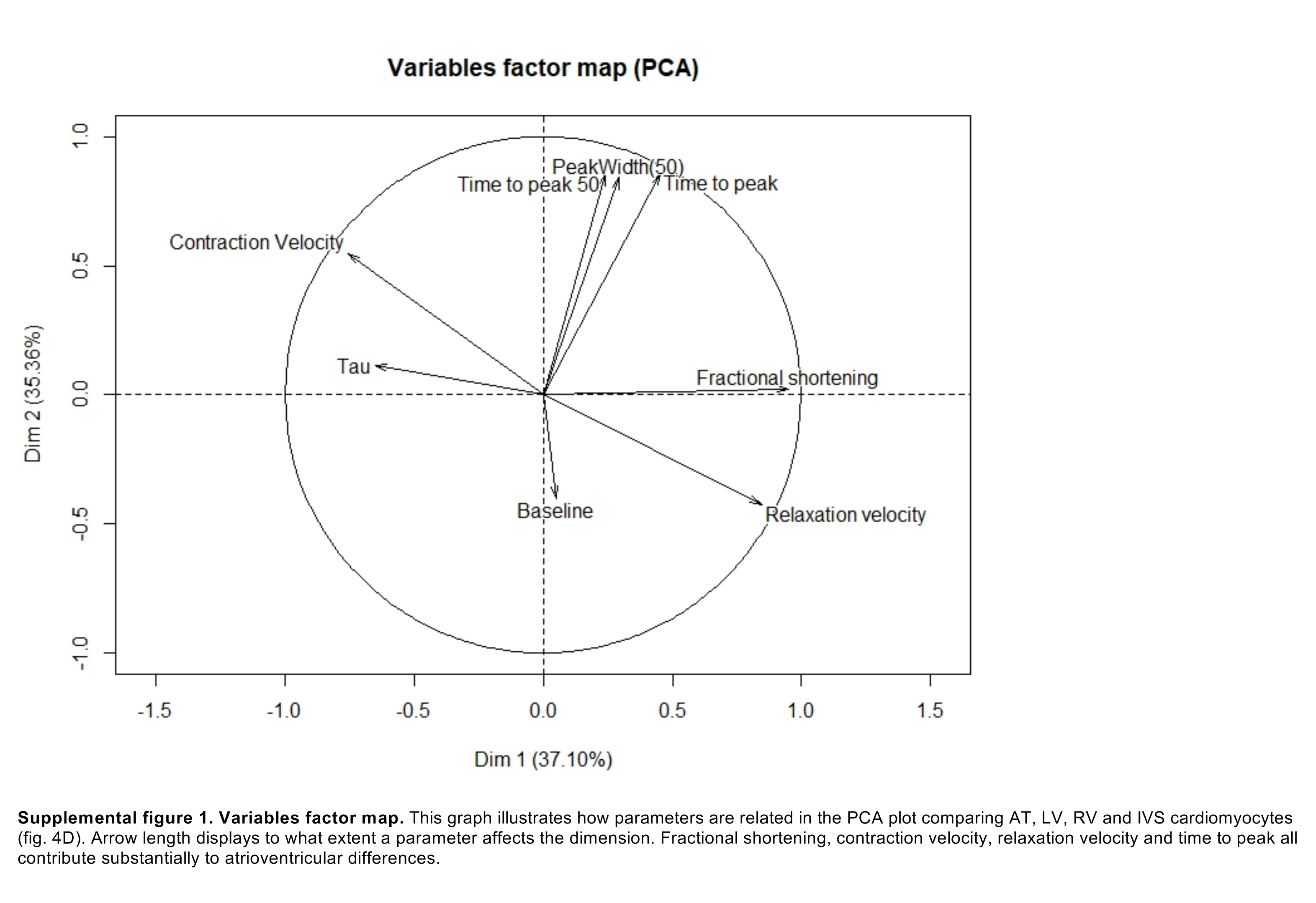

Supplement: Supplementary file 1 [file Image_1.tif]

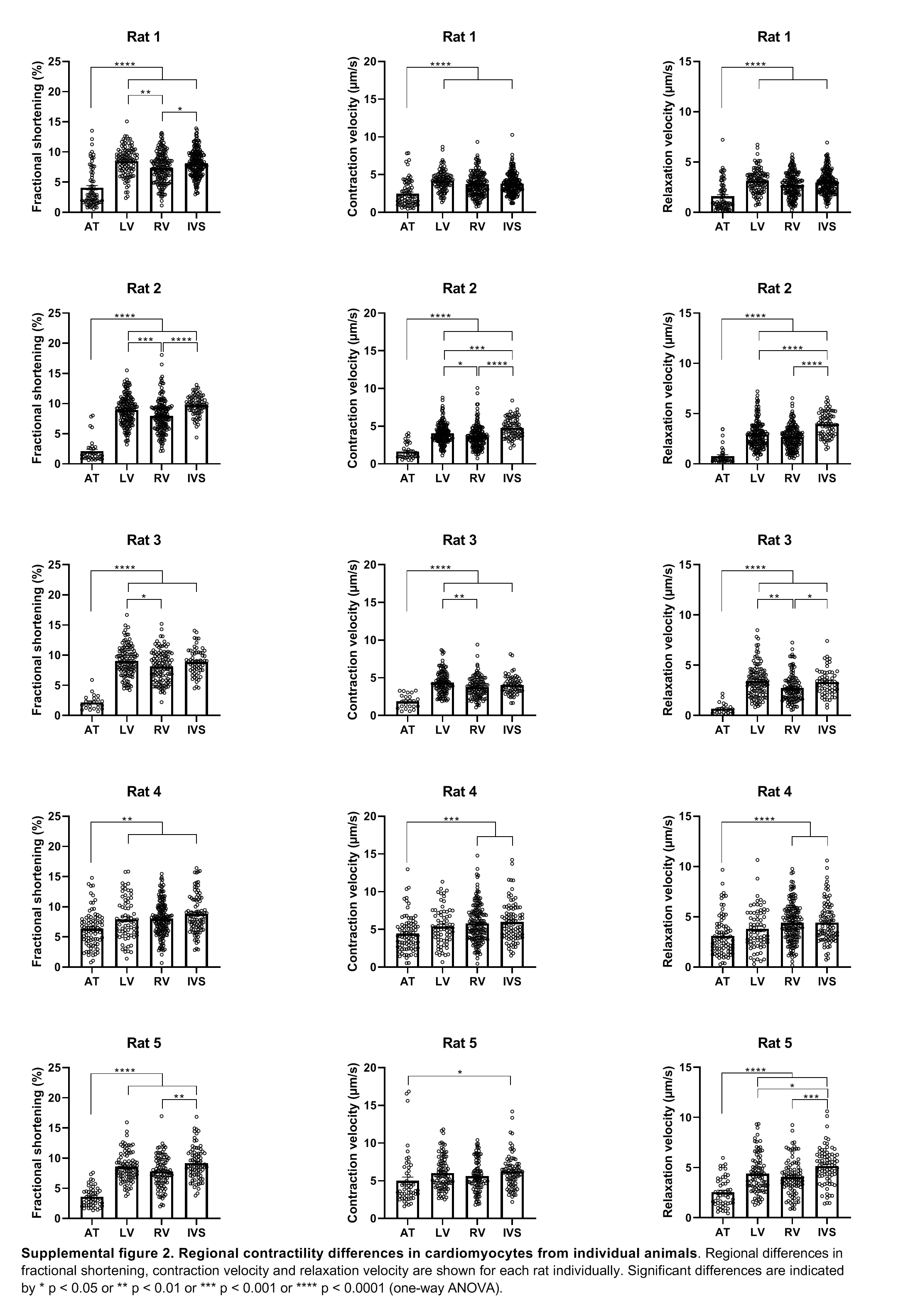

Supplement: Supplementary file 2 [file Image_2.tif]
